# Computational toxicology using the OpenTox application programming interface and Bioclipse

**DOI:** 10.1186/1756-0500-4-487

**Published:** 2011-11-10

**Authors:** Egon L Willighagen, Nina Jeliazkova, Barry Hardy, Roland C Grafström, Ola Spjuth

**Affiliations:** 1 Department of Pharmaceutical Bioinformatics, Uppsala University, Uppsala, Sweden; 2 Division of Molecular Toxicology, Institute of Environmental Medicine, Karolinska Institutet, Stockholm, Sweden; 3 Ideaconsult Ltd, A. Kanchev 4, Sofia 1000, Bulgaria; 4 Douglas Connect, Baermeggenweg 14, 4314 Zeiningen, Switzerland; 5 VTT Technical Research Center of Finland, Medical Biotechnology, FI-20521 Turku, Finland

## Abstract

**Background:**

Toxicity is a complex phenomenon involving the potential adverse effect on a range of biological functions. Predicting toxicity involves using a combination of experimental data (endpoints) and computational methods to generate a set of predictive models. Such models rely strongly on being able to integrate information from many sources. The required integration of biological and chemical information sources requires, however, a common language to express our knowledge ontologically, and interoperating services to build reliable predictive toxicology applications.

**Findings:**

This article describes progress in extending the integrative bio- and cheminformatics platform Bioclipse to interoperate with OpenTox, a semantic web framework which supports open data exchange and toxicology model building. The Bioclipse workbench environment enables functionality from OpenTox web services and easy access to OpenTox resources for evaluating toxicity properties of query molecules. Relevant cases and interfaces based on ten neurotoxins are described to demonstrate the capabilities provided to the user. The integration takes advantage of semantic web technologies, thereby providing an open and simplifying communication standard. Additionally, the use of ontologies ensures proper interoperation and reliable integration of toxicity information from both experimental and computational sources.

**Conclusions:**

A novel computational toxicity assessment platform was generated from integration of two open science platforms related to toxicology: Bioclipse, that combines a rich scriptable and graphical workbench environment for integration of diverse sets of information sources, and OpenTox, a platform for interoperable toxicology data and computational services. The combination provides improved reliability and operability for handling large data sets by the use of the Open Standards from the OpenTox Application Programming Interface. This enables simultaneous access to a variety of distributed predictive toxicology databases, and algorithm and model resources, taking advantage of the Bioclipse workbench handling the technical layers.

## Findings

We here report the establishment of a new interoperable platform for computational toxicology that is able to dynamically discover computational services running the latest predictive algorithms and models, while hiding technicalities by reusing a graphics-oriented workbench for the life sciences. The OECD QSAR ToolBox [1,2] and ToxTree [3,4] are existing softwares that aggregate predictive toxicity models, but do not integrate with other functionality easily, such as online services. Bioclipse, however, is designed to integrate local and remote functionality [5-7]. In this paper we outline how we implemented a new platform, integrating the OpenTox Open Standards [8] and the interactive, but scriptable Open Source workbench for the life sciences, Bioclipse. This approach makes it possible for anyone to make new computational toxicology models available to Bioclipse without the need to change the software source code.

Predictive toxicology is a field where knowledge from many sources needs to be integrated to provide a weight of evidence on the toxicity of untested chemical compounds. Typical sources of information include databases with *in vivo *and *in vitro *experimental data such as ToxCast and SuperToxic [9,10], literature databases summarizing adverse reactions like SIDER [11], and computational resources based on toxicity data for other compounds including DSSTox [12]. Importantly, this information should be visualized, preferably linked to the chemical structure of the compound, or by visualizing relevant life science data, such as gene, protein and biological pathway information [13-15] or metabolic reactions [16]. Bioclipse was designed to provide such interactive data analysis for the life sciences.

Moreover, predictive toxicology is an advancing science, aiming to develop new alternative testing methods, satisfying the demanding risk assessment requirements of the European REACH guidance [17]. The dynamic discovery of new toxicology-related data and computational methods is therefore of utmost scientific and practical importance. The EU FP7 OpenTox project recently developed a framework to enable the feasibility of semantic integration of such new resources [8].

We describe here the subsequent technological interoperation of Bioclipse and the OpenTox platform, such as implemented by the AMBIT software [18]. This short report outlines what functionality the new combined platform provides to the toxicologist and what development is ongoing. At the core of the interoperation lies the use of the Resource Description Framework (RDF) [19] and related Open Standards. OpenTox uses RDF as a primary exchange format and the RDF query language SPARQL [20] to discover data sets, algorithms and models. Bioclipse was recently extended to support these standards [21], simplifying the interoperation task with OpenTox.

We outline three applications that exemplify how the various used technologies make this interoperability possible, starting with a computational toxicology example. Advantage is taken of three technologies that drive the interoperability. First, it uses the SPARQL RDF query language to discover functionality on the OpenTox network. Secondly, it uses the OpenTox web services for remote computation. Finally, all graphical user interfaces use a new Bioclipse Scripting Language (BSL) [6] extension to interact with OpenTox servers, allowing all interaction to be scripted and automated too.

### Computational Toxicology

**Figure 1:**
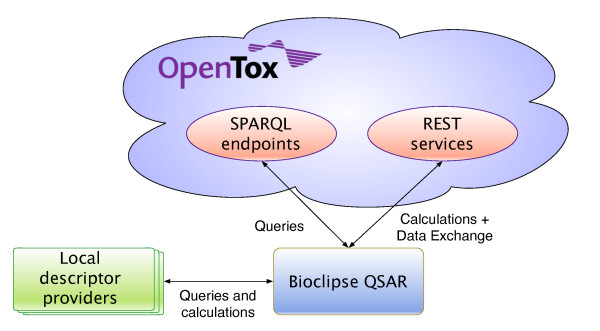
**An overview of the Bioclipse QSAR and OpenTox integration**. Toxicological properties of molecules can be calculated in Bioclipse using the online computational services within the OpenTox cloud, in parallel to local services. When the user calculates these properties, Bioclipse will first query local and online service providers for available functionality. Example services in the OpenTox cloud are the ToxTree toxicology prediction models. The OpenTox cloud is queried by Bioclipse internally using the SPARQL query language. Once the user has selected the toxicological properties of interest (see Figure 2), these will be calculated by Bioclipse. Here, REST technologies are used to perform this computation in the OpenTox cloud. The computed results can then be used in Bioclipse.

Figure 1 shows how the interoperability of Bioclipse with the OpenTox API is designed, and in particular how it was used to extend the molecular descriptor calculation functionality in Bioclipse described previously [22]. This functionality can be used to calculate properties such as logP and pKa, important to various aspects of toxicity, including membrane transport and receptor binding. Knowledge about such properties can be used under the European REACH regulation. For example, predicted physical and chemical properties can, under certain conditions, complement toxicity testing using animal experiments, and as such, calculation of such descriptors is increasingly relevant.

**Figure 2:**
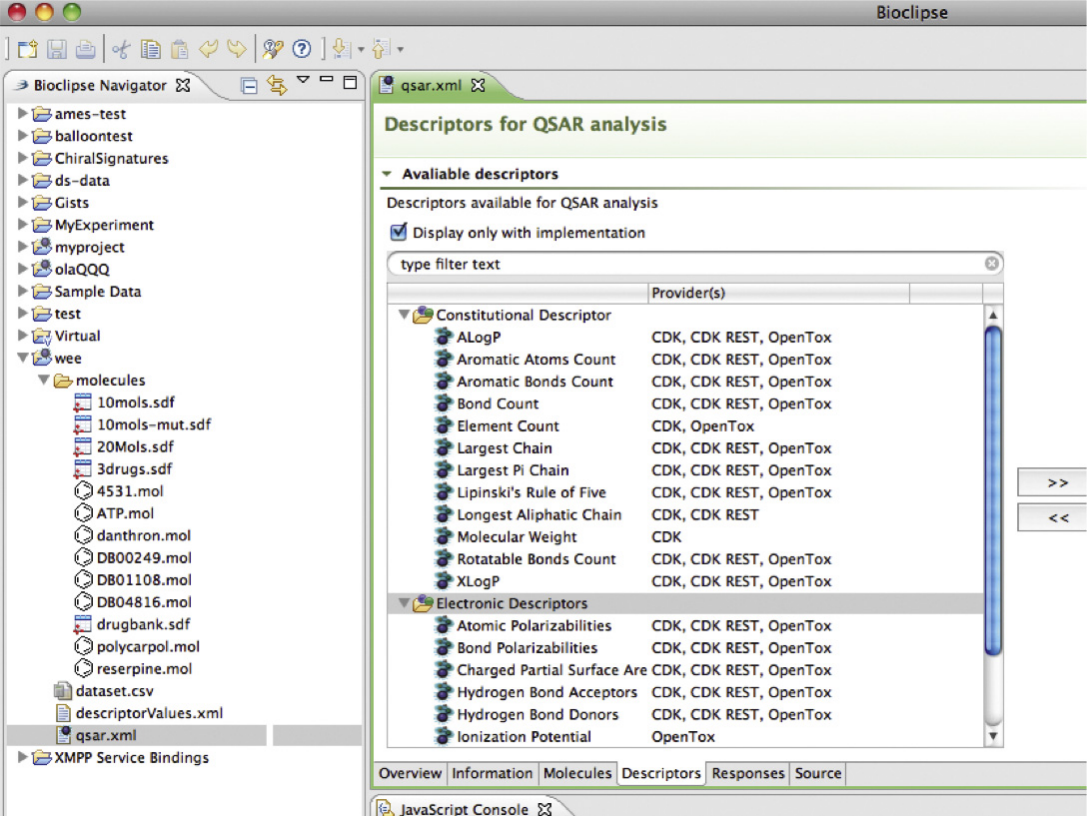
**Integration of OpenTox descriptors in Bioclipse QSAR**. Molecular descriptors are much used in computational toxicology models. This screenshot from Bioclipse QSAR shows descriptors discovered on the Internet (providers: OpenTox and CDK REST) in combination with local software (provider: CDK).

Bioclipse dynamically discovers descriptor algorithms exposed via the OpenTox servers, using the OpenTox ontology service's SPARQL endpoint. This *SPARQL endpoint *functions as a registry of available computational services on the OpenTox network, similar to the role of BioCatalogue [23]. These services are described with the OpenTox ontology, which is available as Web Ontology Language [24] document at http://opentox.org/api/1_1/opentox.owl and discussed in detail in reference [8]. Using the SPARQL query language Bioclipse can retrieve a list of available services. Moreover, when a new descriptor algorithm or model is registered on the OpenTox ontology service, it will automatically be picked up by Bioclipse. Figure 2 shows several discovered OpenTox descriptor algorithms, along with algorithms from other local (*CDK *[25]) and remote (*CDK REST*) providers. Using this approach, Bioclipse has access to the most recent descriptors relevant to toxicity predictions.

OpenTox provides web services to calculate a descriptor value for a given molecule. Using the linked resources idea of the semantic web, the descriptors discovered via the ontology server can be invoked via Bioclipse directly. As such, OpenTox-provided descriptor calculations can be mixed with descriptor calculations local to Bioclipse, or from other remote computational services, as described before [22]. This creates a flexible application for the integration of numerical input for statistical modeling of toxicologically relevant end points, as well as the comparison of various predictive models for a more balanced property analysis.

All functionality for remote computing on the OpenTox network is also available as BSL scripting commands, allowing all OpenTox interoperation with the Bioclipse graphical user interface to be replicated using BSL scripts. Table 1 shows the BSL commands for service and data discovery and the invocation of remote services, under the categories *Querying *and *Computation*, respectively.

**Table 1:** BSL script commands for interacting with the OpenTox platform

Command(parameters)	Description
Querying	

listModels(service)	Lists the predictive models available from the given service.
getFeatureInfo(ontologyServer, feature)	Returns information about a particular molecular feature (property).
getFeatureInfo(ontologyServer, features)	Returns information about a set of molecular features.
getModelInfo(ontologyServer, model)	Returns information for a computational model.
getModelInfo(ontologyServer, models)	Returns information for a list of computational models.
getAlgorithmInfo(ontologyServer, algorithm)	Returns information for a computational algorithm.
getAlgorithmInfo(ontologyServer, algorithms)	Returns information for a list of computational algorithms.
listAlgorithms(ontologyServer)	Returns a list of algorithms.
listDescriptors(ontologyServer)	Returns a list of descriptor algorithms.
listDataSets(service)	Returns the data sets available at the given OpenTox server.
searchDataSets(ontologyServer, query)	Returns matching data sets using a free text search.
search(service, inchi)	Returns matching structures based on the InChI given.
search(service, molecule)	Returns matching structures based on the molecule given.

Computation	

calculateDescriptor(service, descriptor, molecules)	Calculates a descriptor value for a set of molecules.
calculateDescriptor(service, descriptor, molecule)	Calculates a descriptor value for a single molecule.
predictWithModel(service, model, molecules)	Predicts modeled properties for the given list of molecules.
predictWithModel(service, model, molecule)	Predicts modeled properties for the given molecule.

Data exchange	

createDataset(service)	Creates a new data set on an OpenTox server.
createDataset(service, molecules)	Creates a new data set on an OpenTox server and adds the given molecules.
createDataset(service, molecule)	Creates a new data set on an OpenTox server and adds a single molecule.
addMolecule(dataset, mol)	Adds a molecule to an existing data set.
addMolecules(dataset, molecules)	Adds a list of molecules to an existing data set.
deleteDataset(dataset)	Deletes a data set.
downloadCompoundAsMDLMolfile(service, dataset, molecule)	Downloads a molecule from a data set as a MDL molfile.
downloadDataSetAsMDLSDfile(service, dataset, file-name)	Download a complete data set as MDL SD file and saves it to a local file in the Bioclipse workspace.
listCompounds(service, dataset)	Lists the molecules in a data set.

Authentication	

login(accountname, password)	Authenticate the user with OpenSSO and login on the OpenTox network.
logout()	Logout from the OpenTox network.
getToken()	Returns a security token when Bioclipse is logged in on the OpenTox network.

### Data Sharing

**Figure 3:**
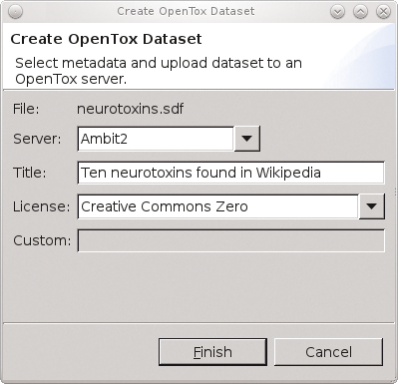
**Graphical user interface for uploading data to OpenTox**. Sharing new toxicological data about molecular structures can be done by uploading the data to an OpenTox server. This Bioclipse dialog shows a select MDL SD file with ten neurotoxins (*neurotoxins.sdf*) being shared on the Ambit2 server, the OpenTox server to upload to, providing a title for the data set, and a license (see main text). Clicking the Finish button will upload the structures and open a web browser window in Bioclipse with the resulting online data set (see Figure 4).

Using a second, data sharing use case we will explain how all graphical interoperation is using a BSL script extension. For example, Figure 3 shows the Bioclipse dialog for uploading a small data set with ten neurotoxins to an OpenTox server (see Additional file 1). This dialog asks which OpenTox server to upload to (the *Ambit2 *server is selected, http://apps.ideaconsult.net:8080/ambit2/), a title under which this data set will be available ("Ten neurotoxins found in Wikipedia"), and the data license or waiver under which the data will be available to others. Figure 3 indicates that the Creative Commons Zero waiver [26] was selected. Other options include the ODC Public Domain Dedication and Licence [27], Open Database License [28], and the Open Data Commons Attribution License [29]. Optionally, the user can specify a web location for a custom license agreement under which the data is available, though we encourage users to select a standard license.

**Figure 4:**
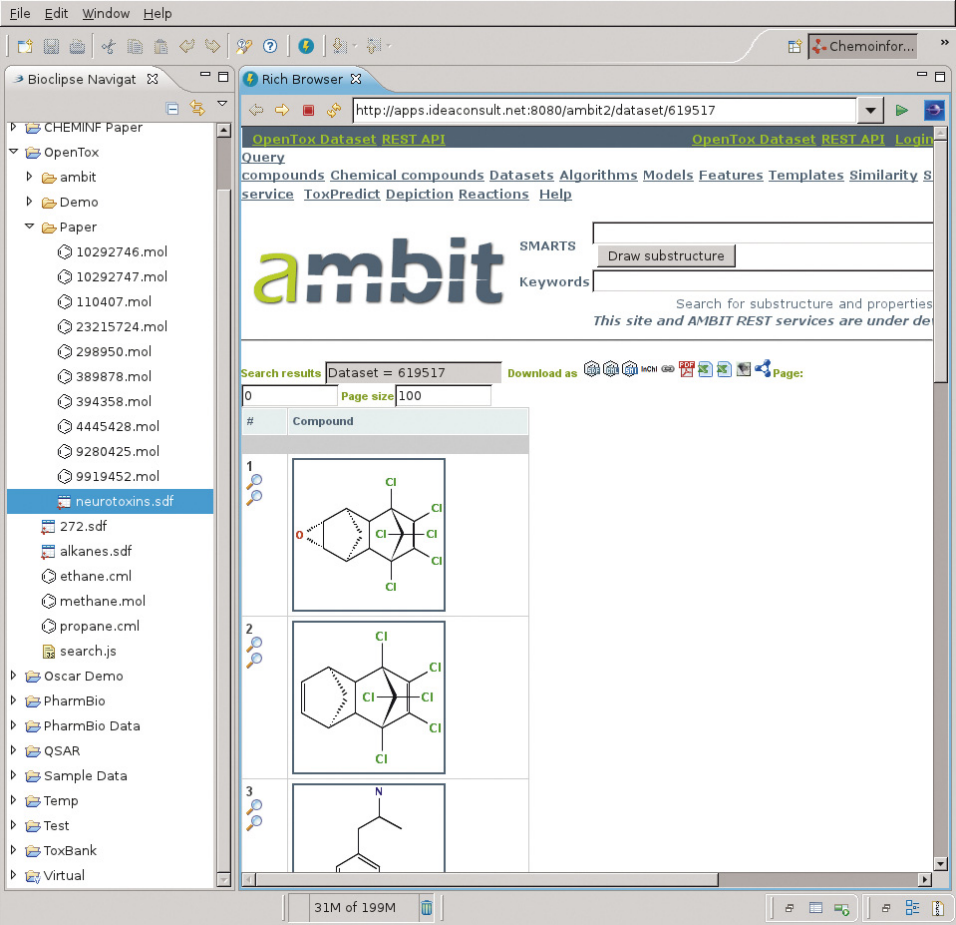
**OpenTox web page showing uploaded data**. Screenshot of Bioclipse showing a web browser window with the neurotoxins data hosted on the Ambit2 OpenTox server after the upload, as shown in Figure 3 (see http://apps.ideaconsult.net:8080/ambit2/dataset/619517).

Technically, the dialog makes use of the script commands *createDataSet *(*service, molecules*), *setDatasetLicense *(*datasetURI, licenseURI*), and *setDatasetTitle *(*datasetURI, title*) (see Table 1). The latter two methods use the data set Universal Resource Identifier (URI) returned by the first method. When the upload has finished, the resulting OpenTox web page is opened in a browser window in Bioclipse (see Figure 4).

This use case shows nicely how the Bioclipse-OpenTox integration takes advantage of the fact that Bioclipse has all graphical user interface (GUI) functionality matched by a scripted equivalent. The use of the BSL directly, allows interaction with the OpenTox network to be automated, combined with other Bioclipse functionality into larger workflows, and makes it easier to share procedures with others, using social scientific sites like MyExperiment [30]. An example BSL script for calculating molecular descriptors combines OpenTox functionality with cheminformatics functionality provided by the *cdk *script extensions (also available as Additional file. 2):

//requires an unspecified Bioclipse development version

//bioclipse.requireVersion("2.6")

service = "http://apps.ideaconsult.net:8080/ambit2/";

serviceSPARQL = "http://apps.ideaconsult.net:8080/ontology/";

stringMat = opentox.listDescriptors(serviceSPARQL);

stringMat.getColumn("algo");//returns the descriptor services

stringMat.getColumn("desc");//returns the BO entries

descriptor = stringMat.get(1,1);

molecules = cdk.createMoleculeList();

molecules.add(

   cdk.fromSMILES(CC(=O)C1=CC=C(C=C1)N")

);

molecules.add(

   cdk.fromSMILES("C1=CC=C(C(=C1)CC(=O)O)NC2=C(C=CC=C2C1)C1")

);

js.say(

   descriptor + " - " +

   opentox.calculateDescriptor(service, descriptor, molecules)

);

This will generate the following output to the JavaScript console:

http://apps.ideaconsult.net:8080/ambit2/algorithm/org.openscience.cdk.qsar.descriptors.molecular.XLogPDescriptor - [0.11900000274181366, 2.2190001010894775]

Table 1 shows an overview of the available BSL commands for uploading data to and downloading data from OpenTox servers under the heading *Data exchange*.

### Authentication

The third demonstration of Bioclipse-OpenTox interoperability is the support for accessing protected resources within the OpenTox network. Despite preferences of the authors, we acknowledge that not all scientific data will be Open Data. As such, authentication and authorization (A&A) are important features of data access. OpenTox implements both aspects, and provides web services for A&A, allowing users to log in and out of OpenTox applications, accompanied by policy-based specification of OpenTox resource access permissions. Additionally, the same mechanism is used to restrict the access to calculation procedures, allowing to expose software with commercial licenses as protected OpenTox resources. Bioclipse was extended to support the OpenTox authentication, allowing the OpenTox servers to properly authorize the user access to particular web services and data sets. The OpenTox account information is registered with Bioclipse' keyring system, centralizing logging in and out onto remote services, providing the graphical user interface for adding a new OpenTox account and to log in and out. The corresponding script commands for the authentication are given in *Authentication *category in Table 1. Interested people can create a free account at http://www.opentox.org/join_form.

## Discussion

We have described here an interoperability advance, enabling users to interactively explore and evaluate the toxicity properties of molecules based on a semantic web approach to toxicology resources. The integration into Bioclipse makes various components of the OpenTox platform available to the user, both via the graphical user interface as well as via the Bioclipse Scripting Language. The Bioclipse-OpenTox plugin makes it possible to upload data sets to and download them from any OpenTox server, calculate molecular descriptors, and apply predictive toxicology models on molecular structures. All functionality has support for user authentication using the OpenTox-adopted OpenSSO technology. Other components of OpenTox, like model building and validation, have not been added yet, as Bioclipse currently does not have a clear GUI for such functionality yet. Such functionality is being worked on, but outside the scope of this report. The presented aspects make this integration fairly unique; creating a solution which is capable of dynamically discovering new services in the OpenTox network when it starts, which differentiates the software from specialized software like ToxTree and the OECD QSAR ToolBox. These tools aggregate several predictive models, but need to be updated manually by the developers for each new model. However, it is noted that these tools can also be extended to support the OpenTox platform. An added value is that updates to computational modules are only done on the server side, so that the client software (Bioclipse) does not need to be updated; a feature in common with web-based solutions like ToxPredict [31]. The scripting functionality makes it easy to automate data workflows as do workflow applications such as Taverna [32] and KNIME (http://knime.org), but the combination with the rich Bioclipse user interface makes it possible at the same time to work with OpenTox interactively. The calculation results are cached by the OpenTox dataset service, allowing to avoid time consuming processing if the same calculation on the same dataset is requested more than once. Users of the integrated Bioclipse-OpenTox environment do not, therefore, need to care about the performance on their own computer, though we are also exploring the options to have Bioclipse itself run an OpenTox server. The latter is technically possible, and would convert the integrated platform into a standalone application that does not require web access.

From a technological perspective, the Bioclipse-OpenTox integration relies on semantic web technologies, which are seeing significant adoption in other areas of the life sciences too, including drug discovery, text mining, and neurosciences [33-35]. The OpenTox platform demonstrated the provision of a simple but well-defined and consistent ontology for the interaction with their services, providing functionality for both service discovery and service invocation. The SADI framework is the only known semantic alternative [36], but does currently not provide the same level of computational toxicology services as OpenTox does. However, while the integration is greatly simplified and semantically defines what services are available and do, the used technologies do neither solve the problem of the chemical validity of the molecular structures that are sent around, nor does it semantically define and specify in detail how to interpret the computational results of toxicity predictions. The first problem refers to the problem that even with explicit meaning we can make incorrect claims. For example, we can always define a triple stating that :*water :isToxicAtLowConcentrationsTo :human*, by using ontologies for all aspects, but that would not make it true. Semantic technologies are not about correctness. Instead, they make it much easier to find inconsistencies between knowledge bases. The same argument applies to semantically marked up molecular structures and other data passed between Bioclipse and the OpenTox cloud (cf. Figure 1).

An example of the second problem is that various services can indicate that a compound is mutagenic or carcinogenic, but express that statement in different ways. One service may return a binary yes/no answer, while another returns a more detailed answer, such as for which cell line or organism the prediction is made. Such semantic integration is currently outside the scope of this Bioclipse-OpenTox interoperability, but it is not a problem unique to our approach either.

To address these issues, the community needs to develop better capabilities to link automatically and reliably the various concepts in toxicology, such as links between chemical names and structures and links to toxicities based on current biological knowledge on effects, targets and pathways. The platform is ready for such semantic integration, but the community needs to develop a common language, which will be enabled through the creation of a public set of linked, harmonized and interoperable ontologies satisfying the predictive toxicology use cases of the future, supporting an integrated data analysis.

## Availability and requirements

• **Project name**: Bioclipe-OpenTox

• **Project home page**: http://www.bioclipse.net/opentox/

• **Operating system(s)**: Platform independent

• **Programming language**: Java

• **Other requirements**: Java 6 or higher

• **License**: Eclipse Public License

• **Any restrictions to use by non-academics**: None

## List of abbreviations

A&A: Authorization and Authentication; API: Application Programming Interface; BSL: Bioclipse Scripting Language; CDK: Chemistry Development Kit; EU: European Union; FP7: Seventh Framework Programme; GUI: Graphical User Interface; OECD: Organisation for Economic Co-operation and Development; QSAR: Quantitative Structure-Activity Relationship; RDF: Resource Description Framework; REACH: Registration, Evaluation, Authorisation and Restriction of Chemical substances; REST: Representational State Transfer; SPARQL: SPARQL Protocol and RDF Query Language; URI: Uniform Resource Identifier.

## Competing interests

OS declares interest in Genetta Soft AB, Sweden. NJ declares interest in Ideaconsult Ltd., Bulgaria.

## Authors' contributions

EW initiated the project at Uppsala University. OS and EW integrated the two platforms. NJ worked on OpenTox to improve internal consistency. BH and RG encouraged and discussed the work with co-authors and users. All authors contributed to the writing of the paper and approved the final version.

## Supplementary Material

Additional file 1 **The structures of ten neurotoxins**. Click here for file

Additional file 2 **Bioclipse Scripting Language script to calculate a molecular descriptor**. Bioclipse Scripting Language script to calculate the first molecular descriptor it finds on the OpenTox server Ambit2 for two structures created from the molecular line notation format SMILES. A similar script is available from MyExperiment at http://www.myexperiment.org/workflows/1646.
Click here for file
